# Evaluating the efficacy of dexamethasone in the treatment of patients with persistent acute respiratory distress syndrome: study protocol for a randomized controlled trial

**DOI:** 10.1186/s13063-016-1456-4

**Published:** 2016-07-22

**Authors:** Jesús Villar, Javier Belda, José Manuel Añón, Jesús Blanco, Lina Pérez-Méndez, Carlos Ferrando, Domingo Martínez, Juan Alfonso Soler, Alfonso Ambrós, Tomás Muñoz, Rosana Rivas, Ruth Corpas, Francisco J. Díaz-Dominguez, Marina Soro, Miguel Angel García-Bello, Rosa Lidia Fernández, Robert M. Kacmarek, the DEXA-ARDS Network

**Affiliations:** CIBER de Enfermedades Respiratorias, Instituto de Salud Carlos III, Madrid, Spain; Multidisciplinary Organ Dysfunction Evaluation Research Network, Research Unit, Hospital Universitario Dr. Negrín, Barranco de la Ballena s/n, 4th floor – South Wing, 35019 Las Palmas de Gran Canaria, Spain; Keenan Research Center for Biomedical Science at the Li Ka Shing Knowledge Institute, St. Michael’s Hospital, Toronto, ON Canada; Department of Anesthesiology, Hospital Clínico Universitario de Valencia, Valencia, Spain; Intensive Care Unit, Hospital Virgen de La Luz, Cuenca, Spain; Intensive Care Unit, Hospital Universitario Río Hortega, Valladolid, Spain; Division of Clinical Epidemiology and Biostatistics, Research Unit, Hospital Universitario NS de Candelaria, Santa Cruz de Tenerife, Spain; Intensive Care Unit, Hospital Virgen de la Arrixaca, Murcia, Spain; Intensive Care Unit, Hospital Morales Meseguer, Murcia, Spain; Intensive Care Unit, Hospital General de Ciudad Real, Ciudad Real, Spain; Intensive Care Unit, Hospital Universitario de Cruces, Barakaldo, Vizcaya Spain; Intensive care Unit, Hospital Galdakao-Usansolo, Usansolo, Vizcaya Spain; Intensive Care Unit, Hospital N.S. del Prado, Talavera de la Reina, Toledo, Spain; Intensive Care Unit, Hospital General de León, León, Spain; Biostatistics, Research Unit, Hospital Universitario Dr. Negrín, Las Palmas, Spain; Department of Respiratory Care, Massachusetts General Hospital, Boston, MA USA; Department of Anesthesiology, Harvard University, Boston, MA USA

**Keywords:** Acute respiratory distress syndrome, Dexamethasone, Corticoids, Positive end-expiratory pressure, Lung-protective ventilation

## Abstract

**Background:**

Although much has evolved in our understanding of the pathogenesis and factors affecting outcome of patients with acute respiratory distress syndrome (ARDS), still there is no specific pharmacologic treatment for ARDS. Several clinical trials have evaluated the utility of corticoids but none of them has demonstrated a definitive benefit due to small sample sizes, selection bias, patient heterogeneity, and time of initiation of treatment or duration of therapy. We postulated that adjunctive treatment of persistent ARDS with intravenous dexamethasone might change the pulmonary and systemic inflammatory response and thereby reduce morbidity, leading to a decrease in duration of mechanical ventilation and a decrease in mortality.

**Methods/design:**

This is a prospective, multicenter, randomized, controlled trial in 314 patients with persistent moderate/severe ARDS. Persistent ARDS is defined as maintaining a PaO_2_/FiO_2_ ≤ 200 mmHg on PEEP ≥ 10 cmH_2_O and FiO_2_ ≥ 0.5 after 24 hours of routine intensive care. Eligible patients will be randomly allocated to two arms: (i) conventional treatment without dexamethasone, (ii) conventional treatment plus dexamethasone. Patients in the dexamethasone group will be treated with a daily dose of 20 mg iv from day 1 to day 5, and 10 mg iv from day 6 to day 10. Primary outcome is the number of ventilator-free days, defined as days alive and free from mechanical ventilation at day 28 after intubation. Secondary outcome is all-cause mortality at day 60 after enrollment.

**Discussion:**

This study will be the largest randomized controlled clinical trial to assess the role of dexamethasone in patients with persistent ARDS.

**Trial registration:**

Registered on 21 November 2012 as DEXA-ARDS at ClinicalTrials.gov website (NCT01731795).

**Electronic supplementary material:**

The online version of this article (doi:10.1186/s13063-016-1456-4) contains supplementary material, which is available to authorized users.

## Background

The acute respiratory distress syndrome (ARDS) is an inflammatory disease process of the lungs as a response to both direct and indirect insults, characterized clinically by severe hypoxemia, reduced lung compliance, and bilateral radiographic infiltrates [1]. The mechanisms by which a wide variety of insults can lead to this syndrome are not clear. Although much has evolved in our understanding of its pathogenesis and factors affecting patient outcome, still there is no specific pharmacologic treatment for ARDS. Despite advances in supportive measures, ARDS has a mortality rate of about 40–50 % in most series [2]. Patients with ARDS invariably require mechanical ventilation (MV) to decrease the work of breathing and to improve oxygen transport. To date the only proven, widely accepted method of MV for ARDS is “lung-protective ventilation” using a low tidal volume (V_T_) strategy plus moderate to high levels of positive end-expiratory pressure (PEEP).

Corticoids seemed to be an ideal therapy for the acute lung injury in ARDS, given their potent anti-inflammatory and antifibrotic properties [3]. They switch off genes that encode pro-inflammatory cytokines and switch on genes that encode anti-inflammatory cytokines. It has been reported that low doses of corticosteroids prevent an extended cytokine response and might accelerate the resolution of pulmonary and systemic inflammation in pneumonia [4]. Several clinical trials have evaluated the utility of methylprednisolone in ARDS [5–15]. None of them has demonstrated a definitive benefit due to small sample sizes, selection bias, patient heterogeneity, and time of initiation of treatment or duration of therapy. The ARDS Network performed the largest randomized trial of methylprednisolone versus placebo in 180 patients with ARDS of at least 7 days duration [13]. Although there was no survival benefit in the steroid group, methylprednisolone increased the number of ventilator-free days (VFDs) and intensive care unit (ICU)-free days during the first month. Also, a meta-analysis of selected trials showed that prolonged administration of systemic steroids is associated with favorable outcomes and survival benefit when given before day 14 of ARDS [16].

Despite these disappointing trial results, great interest remains in the use of corticosteroids for the salvage of ARDS patients in the early phase of their disease process, a situation that has not been evaluated in most published trials. Paradoxically, these hormones are given as adjunctive therapy in patients with septic shock [17]. We postulated that adjunctive treatment of persistent ARDS with dexamethasone might change the pulmonary and systemic inflammatory response and thereby reduce morbidity, leading to a decrease in duration of MV and to a decrease in mortality.

## Methods/design

### Justification of the study

Currently, there is no proven pharmacologic treatment for patients with ARDS. Protective MV is the most important aspect of supportive care of ARDS patients. Dexamethasone has never been evaluated in ARDS in a randomized controlled fashion. However, dexamethasone has potent anti-inflammatory effects and weak mineralocorticoid effects compared with other corticosteroids [18]. It is 20 to 30 times more potent than the naturally occurring hormone cortisol and four to five times more potent than prednisone [3]. Dexamethasone has a long-lasting effect, allowing for a once-a-day regimen [18]. Whether addition of dexamethasone to conventional supportive treatment benefits ARDS patients is unknown, it has been used in patients with pneumonia [18], septic shock [19], meningitis [20], and other causes of ARDS [21].

We justify the need of our study based on the positive results of two recent clinical trials: (i) Meijvis et al. [18] showed that dexamethasone (5 mg/day) for 4 days was able to reduce length of hospital stay in 304 patients with bacterial pneumonia when added to conventional treatment; (ii) Azoulay et al. [21] showed that dexamethasone (10 mg/6 h), when added to chemotherapy and conventional ICU management, caused less respiratory deterioration and lower ICU mortality in 40 patients with acute lung injury resulting from leukemia. Our goal is to examine the effects of dexamethasone on length of MV (assessed by number of ventilator-free days) and on 60-day mortality, in patients admitted into a network of Spanish intensive care units (ICUs) who still meet ARDS criteria at 24 hours after ARDS onset despite routine intensive care management.

### Study design

The DEXA-ARDS study is a prospective, multicenter, randomized, controlled trial in 314 patients with persistent ARDS admitted into a network of 18 ICUs from university and community hospitals in Spain (Appendix 1).

The trial has been designed in accordance with the fundamental principles established in the Declaration of Helsinki, the Convention of the European Council related to human rights and biomedicine, and the Universal Declaration of UNESCO on the human genome and human rights, and within the requirements established by Spanish legislation in the field of biomedical research, the protection of personal data, and bioethics, which was classified by the Spanish Agency of Drugs and Medical Devices (Agencia Española del Medicamento y Productos Sanitarios) as a clinical randomized study with drugs on 21 November 2012 and registered on 21 November 2012 at http:/www.clinicaltrials.gov with identification no. NCT01731795. The study was approved by the referral Ethics Committee (Hospital Clínico Universitario de Valencia, Valencia, Spain) and the institutional review boards of all participating hospitals (Additional file 1). For inclusion into the study, signed written informed consent from the patient or the patient’s personal legal representative will be provided (Additional file 2). See Additional file 3 for the SPIRIT checklist of the study protocol.

### Study population

To be eligible for inclusion into this study (day 0), each patient (male or female) must fulfill the following inclusion criteria during screening and prior to enrollment into the study: age ≥18 years, be intubated and mechanically ventilated, and have acute onset of ARDS, as defined by the American-European Consensus Conference (AECC) criteria for ARDS [22] or as moderate/severe ARDS by the Berlin criteria [23], which include: (i) having an initiating clinical condition (pneumonia, aspiration, inhalation injury, sepsis, trauma, acute pancreatitis, etc.); bilateral infiltrates on frontal chest radiograph, (ii) absence of left atrial hypertension, a pulmonary capillary wedge pressure less than 18 mmHg, or no clinical signs of left heart failure, (iii) hypoxemia (as defined by a ratio between partial pressure of oxygen in arterial blood and fraction of inspired oxygen (PaO_2_/FIO_2_) ≤ 200 mmHg on PEEP ≥ 5 cmH_2_O, regardless of FiO_2_). Patients will be excluded from study participation if any of the following are present: pregnancy or active lactation, enrollment in another experimental treatment protocol, brain death, terminal-stage cancer or other terminal disease, having do-not-resuscitate orders, immunocompromised, receiving corticosteroids or immunosuppressive drugs, more than 24 hours elapsed after initially meeting the ARDS criteria before consent and results of initial standard ventilator settings could be obtained, severe chronic obstructive pulmonary disease, or congestive heart failure.

### Enrollment into the study and randomization

For the present study, onset of ARDS was defined as the day and time in which the patient first met ARDS criteria [22, 23]. Screened patients will be considered for enrollment immediately prior to randomization and to the first dose of study medication on day 1 if they still meet the ARDS inclusion criteria at 24 hours after ARDS onset, assessed on standard ventilator settings. Arterial blood gases and hemodynamic and data will be obtained on the following mandatory standard ventilator settings: volume assist/control mode, V_T_ 7 ml/kg predicted body weight (PBW), inspiratory:expiratory (I:E) time ratio 1:1, ventilator rate to maintain a partial pressure of carbon dioxide (PaCO_2_) of 35 to 50 mm Hg, on FiO_2_ ≥ 0.5 and PEEP ≥ 10 cmH_2_O) [24]. If under these ventilator settings, the PaO_2_/FiO_2_ is ≤ 200 mmHg, the patient will be eligible for randomization.

Eligible, informed consented patients will be randomly allocated to two arms: (i) conventional treatment without dexamethasone and (ii) conventional treatment plus dexamethasone. Randomization will be performed by blocks of ten opaque, sealed envelopes sent to each participating ICU, according to a computer-generated random-number table. Randomization will be based on one-to-one allocation of prenumbered envelopes. As requested, a second block of ten sealed envelopes will be sent to those participating ICUs with high enrollment rates.

### Ventilatory management and dexamethasone therapy

**Fig. 1:**
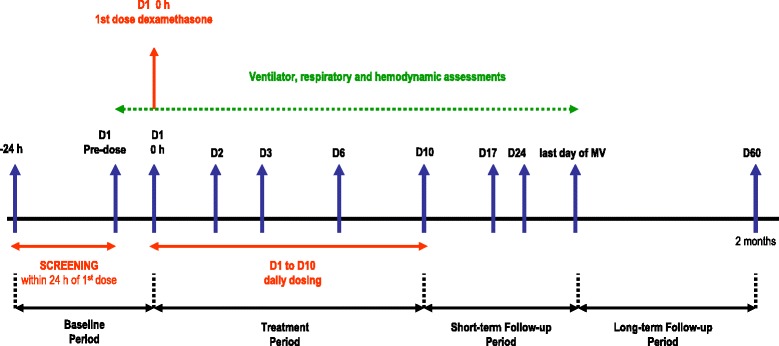
Study design diagram

Both treatment arms will follow current guidelines for supportive care, including antibiotic therapy. Although patient care is not strictly protocolized, physicians are asked to follow current standards for critical care management. For ventilatory management, enrolled patients in both arms will be ventilated with a V_T_ of 4 to 8 ml/kg PBW, a plateau pressure < 30 cmH_2_O, at a respiratory rate to maintain PaCO_2_ 35 to 50 mmHg and PEEP and FiO_2_ combinations that maintain a PaO_2_ > 60 mmHg or a peripheral capillary oxygen saturation (SpO_2_) > 90 %. Patients in the dexamethasone group will be treated with a daily dose of 20 mg iv from day 1 to day 5 and 10 mg iv from day 6 to day 10. We have selected this dose and time of treatment by doubling the dose used by Meijvis et al [18], since our patients are sicker, and half the dose used by Azoulay et al. [21] to avoid development of neutropenia. Our therapeutic regime is dose-equivalent to that from the Steinberg et al trial [13] with methylprednisolone. Treatment with dexamethasone will be maintained for a maximum of 10 days or until extubation (if occurring before day 10 after study enrollment) (Fig. 1).

For weaning from MV in both groups, patients will be assessed daily for readiness for a spontaneous breathing trial based on the ARDSNet protocol [25].

### General procedures

**Fig. 2:**
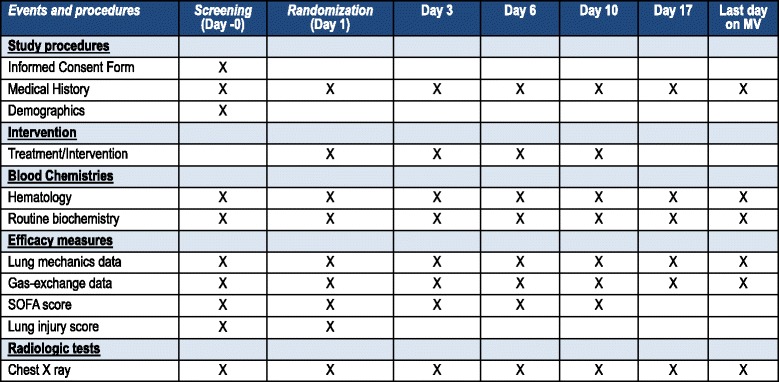
Schedule of events

All participating patients, regardless of the study arm into which they are randomized, will be monitored and managed following general standard of care practices aimed at maintaining optimal conditions. Data from lung mechanics, gas exchange, and hemodynamics will be collected on days 0, 1, 3, 6, and 10, and every 7 days, including the last day of MV (Fig. 2). On each day, the lowest and the highest values of each of the following parameters will be recorded: (i) MV and gas-exchange parameters, including V_T_ (ml/kg PBW), respiratory rate, plateau pressure, PEEP, PaO_2_/FiO_2_, PaCO_2_; (ii) hemodynamics, including blood pressure, heart rate, need for vasoactive drugs; (iii) safety, including frequency of complications (barotrauma, sepsis, pneumonia, etc.), routine biochemistry and hematological tests; (iv) critical care severity scores recorded on days 0 and 1, such as acute physiology and chronic health evaluation II (APACHE II) score [26] and lung injury severity score [27]; (v) total number of extrapulmonary organ failures included in the sequential organ failure assessment (SOFA) scale [28] will be documented daily. Any organ failure occurring during the 6-hour period before death will be considered part of the terminal event and not counted [29]. We will use standard definitions for sepsis and organ failures [28, 30, 31]. We will also monitor ICU and hospital mortality, and duration of MV. Patients will be followed up for 60 days after enrollment.

### Primary and secondary outcomes

The primary outcome of interest is the number of ventilator-free days (VFDs) at 28 days [32], defined as days alive and free from MV at day 28 after intubation. For subjects ventilated ≥ 28 days and for subjects who die, VFD is 0. The secondary outcome of interest is all-cause 60-day mortality.

### Sample size calculations and interim analysis

**Fig. 3:**
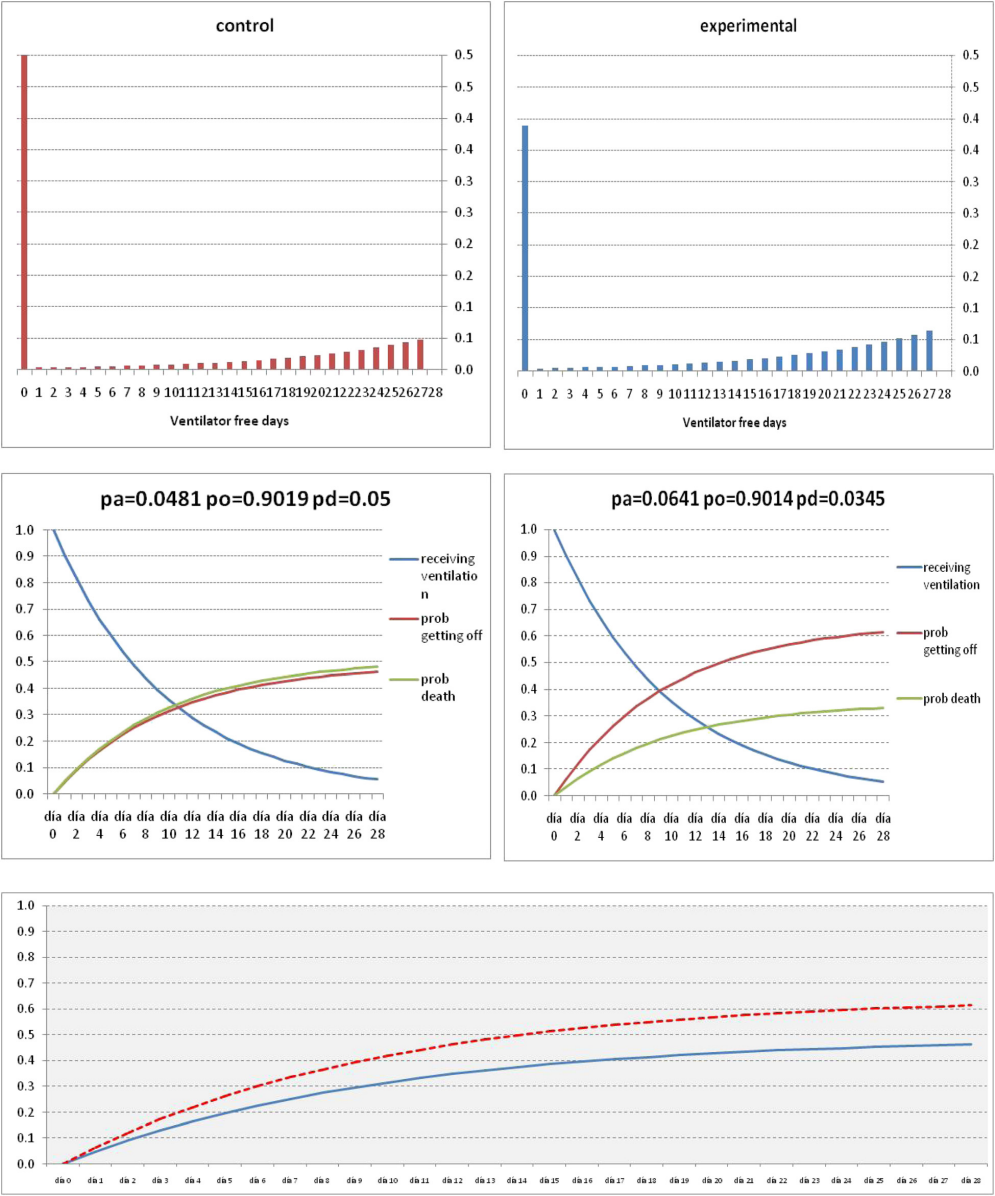
Estimation of samples size calculations based on expected ventilator-free days and mortality. Power analysis has been performed according to Schoenfeld et al [32]. Since we need three probabilities (see Table 1, text highlighted in *yellow*), we have selected three probabilities compatible with our hypothesis (using Mann-Whitney test). We hypothesized that patients in the experimental treatment group have a greater probability of being weaned earlier from the ventilator (0.0641 vs. 0.481) and a lower probability of dying (0.0345 vs. 0.05) for each day of the follow-up period

Since there are no previous pharmacologic studies in patients with persistent ARDS, we estimated the sample size on the assumption that dexamethasone could increase VFDs by ≥ 2 days (from an estimated reference VFDs of 9 days), or would reduce overall 60-day mortality by ≥ 15 % (from a reference control mortality of 50 %). Depending on the estimated standard deviation for the mean VFDs and the expected 60-day mortality rate, we studied various group-size scenarios with cohorts of 147 to 157 patients in each arm to detect these differences with a power of 80 % and a type 1 error of 5 % (two-sided). A population size of 157 patients for each arm (a total of 314 patients) will satisfy all scenarios. No patient loss has been considered. We will only analyze patients that are enrolled and randomized. The power analysis has been performed according to Schoenfeld et al [32] (Fig. 3). Since we need three probabilities for estimating VFD and sample size (Table 1, highlighted in *yellow*), we selected three probabilities compatible with our hypothesis (using the Mann-Whitney test).

**Table 1:** Calculations for the sample size

	VFDs		Probability of getting off ventilation alive	Probability of death	Probability of receiving ventilation	Expected mortality
	Mean	SD	pa	pd	po	60-day mortality
Control group	9.0	10.7	0.0481	0.0500	0.9019	0.4814
Experimental group	12.0	10.8	0.0641	0.0345	0.9014	0.3308
Difference	3.0		0.0160	-0.0155	-0.0005	-0.1506

*VFD* ventilator-free days, *SD* standard deviation

There will be one single formal interim analysis for both efficacy and futility. This analysis will be performed once 50 % of the planned patients have been randomized and initiated treatment and have been followed up to day 60. To protect against an obvious lack of efficacy, failure to achieve an increase of VFDs ≥ 1 day will justify stopping the trial. To protect against a clear improvement of efficacy, achieving a statistically significant difference in VFDs ≥ 3 days or an absolute difference in 60-day mortality ≥ 15 % (two-tailed significant difference *p* < 0.025) will justify stopping the trial. The interim analysis will be performed at the coordinating center (Hospital Universitario Dr. Negrín, Las Palmas de Gran Canaria, Spain) by an external, independent data and safety monitoring board. Investigators enrolling patients will be blinded to the results of this interim analysis if the stopping rule is not applied.

### Statistical analysis of data

Data will be collected in each participating ICU using a standardized form. Data will be transmitted to the coordinating center whenever a patient dies or is discharged from the hospital. Before exporting the data into a computerized database at the coordinating center, a trained data collector from the coordinating center will check the completeness and the quality of information. Logical checks will be performed for missing data and to find inconsistencies, especially regarding clinical diagnosis, date, and severity scores. If necessary, the data collector will contact the investigator by phone to validate the data or reformat the data for entry into the database.

The primary endpoint is the number of ventilator-free days (VFDs) to day 28 after intubation, where all deaths will be awarded zero days. The analysis of VFDs will be performed as intention to treat. By definition, all patients will be mechanically ventilated at the time of ARDS onset. Secondary endpoint is mortality at 60 days (the patient could die before that time period, or could be alive in the ICU, in the hospital or at home). Other outcome endpoints include pulmonary complications (barotrauma), duration of MV, number of extrapulmonary organ failures, and hospital mortality.

Descriptive statistics will be expressed as mean ± SD or median and interquartile range, depending on the nature and distribution of the variables. Inferential statistics will use estimates of the mean of the differences and their 95 % confidence intervals. Variables normally distributed will be compared with the Student’s *t* test. For variables without a normal distribution, the Mann-Whitney *U* rank test will be used for comparison. Categorical variables will be compared using Fisher’s exact test. Probability of survival at 60 days after randomization will be analyzed according to the Kaplan-Meier method, and the results compared with the log-rank test. The relative risks and their 95 % confidence intervals will be estimated. For all these comparisons, we will consider a difference to be statistically significant if *p* < 0.05 (two-sided).

### Trial organization

The steering committee is constituted by the study principal investigators who contributed to its design and approved the final protocol (Appendix 2). The executive committee comprises the main investigators of each participating center and is responsible for administrative, trial, and data management. The data and safety monitoring board is composed of external, independent experts in critical care medicine, mechanical ventilation and ARDS, and with the general data provided by three internal members, it will recommend the continuation or discontinuation of the trial based on the data from the interim analysis (Appendix 2). The trial management team comprises a chief investigator, a project manager, a statistician, a clinical epidemiologist, and an investigator expert in clinical trials. The responsibilities of this team are:

(i) Planning and conducting the study: designing the protocol, case report forms, designing the investigator manual, and managing and controlling the data quality.
(ii) Research center support: assisting the centers with the administrative submission, monitoring recruitment rates, providing sealed randomization envelopes, taking actions to increase patient enrollment, monitoring follow-up, auditing, and sending study materials to the research centers.
(iii) Producing a monthly study newsletter (Dexanews).
(iv) Programming a research-in-progress meeting every 6 months with principal investigators from all sites.
(v) Statistical analysis and research reporting: interim and complete statistical analysis and helping in writing the final manuscript.

## Discussion

This is the first randomized controlled trial designed to evaluate the efficacy of dexamethasone in patients with persistent ARDS and managed with a protective ventilatory strategy, which includes the use of low V_T_, application of moderate to high levels of PEEP, and limitation of the plateau pressures below 30 cmH_2_O.

Corticosteroids have been the most widely used medications for ARDS since the first clinical description of the syndrome [33]. However, after 50 years of intense research, the impact of corticosteroids on ARDS prognosis remains controversial despite numerous observational studies and randomized controlled trials. Published studies exhibit strong publication bias, report inconsistent results and contain a strong heterogeneity due to the inclusion of a wide variety of disease entities and severities and the lack of an early standardized criteria for assessment of lung severity at the time of patient enrollment [34]. Current clinical practice guidelines for ARDS do not recommend corticosteroid therapy, although corticosteroids are given for many reasons, particularly in such potentially fatal conditions as severe pneumonia [35]. The main adverse effect of corticosteroid therapy seems to be hyperglycemia. With the dose and period of treatment suggested in this trial, it will be extremely rare that the prevalence of dexamethasone-induced hyperglycemia would be greater than the associated prevalence of hyperglycemia seen in the acute phases of critical illness [36]. Although the potential disadvantages of corticosteroids should be weighed against the potential benefits, none of the trials performed to date in critically ill patients has shown that the treatment with corticosteroids is harmful [37].

There are major differences between our study and other randomized controlled trials evaluating the impact of corticosteroids in patients with ARDS. First, all the trials published before 2005 evaluated the use of steroids in patients treated with nonprotective MV [5, 7, 8, 10–12]. Second, none of the trials has used the same timing, dosage and type of corticosteroids. Third, none of the trials has specifically evaluated the use of dexamethasone in ARDS. Fourth, none of the trials has consistently reassessed patients at 24 hours after ARDS onset to ensure that only patients with early established ARDS were randomized. As a result, those previous studies enrolled patients with less or greater lung injury but well beyond the 48 hours of the disease process. It has been shown that ARDS is characterized by an overwhelming pulmonary and systemic inflammatory response within 48 hours resulting in exacerbated pulmonary inflammation and fibroproliferation [38]. Failure to repair tissue damage during the first 48 hours results in an ongoing, self-perpetuating inflammation with subsequent loss of lung function and associated increased mortality rate. In our trial, we will ensure that all enrolled patients have established moderate/severe ARDS after 24 hours of meeting the AECC/Berlin definition while on standard ventilator settings. Therefore, the cohort of patients that we will study is different from those studies by the ARDSNet [13] and others [5–8, 10–15]. Finally, although a recent analysis of individual data from four randomized controlled trials with a trial-level meta-analysis [39] showed that early and prolonged corticoid treatment accelerated resolution of ARDS and decreased hospital mortality, none of the individual trials reported in that and other meta-analyses is able to confirm the favorable impact of corticosteroids therapy on the overall mortality of ARDS [16, 34, 39]. Ideally, our study is an adequately powered randomized trial to definitively answer this question.

It should be emphasized that since no studies have been performed to date examining the potential beneficial effects of dexamethasone in ARDS, we only can speculate about the potential limitations of the study. First, if our hypothesis is supported, we cannot generalize our findings to all patients with ARDS. Second, our study design will not allow us to conclude whether administration of dexamethasone for longer or shorter period of time would have the same effects. Third, our study design will not allow us to conclude whether a different dosage would have different effects. Also, since no studies have been performed to date examining the potential beneficial effects of dexamethasone in ARDS, we only can speculate about the potential strengths of the study. First, if our hypothesis is correct, this study will mark an inflection point in the treatment of patients with severe acute lung injury. Second, if our hypothesis is correct, it will be the first time that treatment with a well-known anti-inflammatory drug, such as dexamethasone, will decrease morbidity and mortality of patients with established ARDS. If our hypothesis is correct, expected benefits for public health will include: earlier “liberation” of patient from MV; less probability of occurrence of ICU-associated “dangerous and/or lethal” complications (shock, sepsis, pneumonia, multiple system organ failure); earlier discharge from the ICU; earlier discharge from the hospital, and marked reduction of health care cost.

### Trial status

The first patient was enrolled on 28 March 2013. Expected duration of the study is 50 months.

## Abbreviations

AECC, American-European Consensus Conference; APACHE II, acute physiology and chronic health evaluation II; ARDS, acute respiratory distress syndrome; ICU, intensive care unit; MV, mechanical ventilation; PaCO_2_, partial pressure of carbon dioxide; PaO_2_/FiO_2_, ratio between partial pressure of oxygen in arterial blood and fraction of inspired oxygen; PBW, predicted body weight; PEEP, positive end-expiratory pressure; SOFA, sequential organ failure assessment; SpO_2_, peripheral capillary oxygen saturation; VFD, ventilator-free days; V_T_, tidal volume

## Appendix 1

Steering committee: Jesús Villar, Javier Belda, José Manuel Añón, Jesús Blanco, Lina Pérez-Méndez, Robert M. Kacmarek.

Data and safety monitoring board: Elizabeth Zavala, Fernando Suárez-Sipmann, Marianela Hernandez (external members); Rosa L. Fernández, Miguel A. García-Bello, Jesús Villar (internal members).

## Appendix 2. DEXA-ARDS Network investigators (in alphabetic order by hospitals)

Fernando Mosteiro, Ana María Díaz-Lamas, Regina Arrojo (Hospital de A Coruña, La Coruña, Spain); Carlos Ferrando, Javier Belda, Marina Soro, José Ferreres, José Blanquer-Olivas (Hospital Clínico de Valencia, Valencia, Spain); Tomás Muñoz (Hospital Universitario de Cruces, Bilbao, Spain); Mario Chico, Darío Toral (Hospital Doce de Octubre, Madrid, Spain); Jesús Villar, Rosa Lidia Fernández, Miguel Ángel García-Bello (Hospital Universitario Dr. Negrin, Las Palmas de Gran Canaria, Spain); Rosana Rivas (Hospital Galdakao-Usansolo, Bilbao, Spain); Alfonso Ambrós, Carmen Martín, Rafael del Campo (Hospital General de Ciudad Real, Ciudad Real, Spain); Demetrio Carriedo, Francisco Javier Díaz, Raúl Ismael González-Luengo (Hospital General de León, León, Spain); Cristina Domínguez, Agustín Cariñena, Julián Álvarez (Hospital General de Santiago de Compostela, La Coruña, Spain); Santiago Macías, Alec Tallet (Hospital General de Segovia, Segovia, Spain); Juan A. Soler, Lucía Capilla (Hospital Morales Meseguer, Murcia, Spain); Lina Pérez-Mendez (Hospital Universitario N.S. de Candelaria, Santa Cruz de Tenerife, Spain); Francisco Alba, Ruth Corpas (Hospital NS del Prado, Talavera de la Reina, Toledo, Spain); Miguel A. Romera (Hospital Universitario Puerta de Hierro, Madrid, Spain); Lorena Fernández, Ana María Prieto, Jesús Blanco (Hospital Universitario Río Hortega, Valladolid, Spain); Domingo Martínez, Luís A. Conesa, Manuel A. García (Hospital Virgen Arrixaca, Murcia, Spain); José M. Añón, Elena González, Rosario Solano (Hospital Virgen de la Luz, Cuenca, Spain); María Mar Cruz, María Ángela Magro (Hospital Virgen de la Salud, Toledo, Spain; Robert M. Kacmarek (Massachusetts General Hospital, Boston, MA, USA).

## Additional files

Additional file 1: Approval of the referral ethics committee and the institutional review boards (IRB) of all participating hospitals. Additional file 2: Informed consent form. (PDF 114 kb) Additional file 3: SPIRIT checklist. (PDF 39 kb)
