# Supplementary material for: Evaluating the efficacy of dexamethasone in the treatment of patients with persistent acute respiratory distress syndrome: study protocol for a randomized controlled trial
Source: Trials. 2016 Jul 22;17:342. doi: 10.1186/s13063-016-1456-4 (PMC4957909; doi:10.1186/s13063-016-1456-4)
Supplement: Additional file 1: — Approval of the referral ethics committee and the institutional review boards (IRB) of all participating hospitals. [file 13063_2016_1456_MOESM1_ESM.pdf]

GENERALITAT VALENCIANA

AGÈNCIA VALENCIANA DE SALUT

DICTAMEN DEL COMITÉ ÉTICO DE INVESTIGACIÓN CLÍNICA

Don Manuel Labiós Gómez, Secretario del Comité Ético de Investigación Clínica del **Hospital Clínico Universitario de Valencia**

CERTIFICA

Que este Comité, actuando en calidad de CEIC de referencia, ha evaluado la propuesta del promotor para que se realice el estudio:

Nº EUDRACT: **2012-000775-17**
Código de protocolo del promotor: **PI11-0143**
Versión/fecha del protocolo: **Versión 3.0 de 3 de Junio de 2012**
Hoja de información al paciente/sujeto (versión/fecha): **Versión 2.0 de 16 de octubre de 2011**
Título**: Estudio comparativo, aleatorio, controlado para evaluar la eficacia dde la administración de dexametasona en el tratamiento de pacientes con el Síndrome de Distrés Respiratorio Agudo**
A comparative, randomised controlled trial for evaluating the efficacy of dexamethasone administration in the treatment of patients with the Acute Respiratory Distress Syndrome
Promotor: **Jesús Villar Hernández**

Los centros de investigadores, incluidos en la solicitud del ensayo clínico son:

Y tomando en consideración las siguientes cuestiones:

- La pertinencia del estudio, teniendo en cuenta el conocimiento disponible, así como los requisitos del Real Decreto 223/2004, de 6 de febrero y las normas que lo desarrollan.
- Los requisitos necesarios de idoneidad del protocolo en relación con los objetivos del estudio, justificación de los riesgos y molestias previsibles para el sujeto, así como los beneficios esperados.
- El seguro o la garantía financiera previstos.
- El procedimiento para obtener el consentimiento informado, incluyendo la hoja de información para los sujetos, el plan de reclutamiento de sujetos y las compensaciones previstas para los sujetos por daños que pudieran derivarse de su participación en el ensayo.
- El alcance de las compensaciones económicas previstas y su posible interferencia con el respeto a los postulados éticos.

Este Comité, habiendo tenido en cuenta los informes recibidos de los CEIC implicados, emite un DICTAMEN FAVORABLE para la realización de dicho ensayo en España en los centros que se listan a continuación:

- Hospital Clínico Universitario de Valencia, por el Dr. Fco. Javier Belda.
- Hospital General Virgen de la Luz, por el Dr. José M. Añón.
- Hospital Universitario Rio Hortega, por el Dr. Jesús Blanca.
- Complejo Hospitalario Universitario de Santiago, por el Dr. Antonio Santos.
- Hospital José M. Morales Meseguer, por el Dr. Gumersindo González.
- Complejo Asistencial Universitario de León, por el Dr. Demetrio Carriedo.
- Complexo Hospitalario Universitario a Coruña, por el Dr. Fernando Mosteiro.

- Hospital General de Ciudad Real, por el Dr. Alfonso Ambrós.
- Hospital General La Mancha Centro, por la Dra. Carmen Martín.
- Hospital Universitari Mutua de Terrassa, por la Dra. Mª Mar Fernández.
- Hospital Moncloa, por el Dr. Vicente Gómez.
- Hospital Nuestra Señora de la Candelaria, por la Dra. Elena Espinosa.
- Hospital Universitario de Cruces, por el Dr. Fermín Labayen.
- Hospital General de Segovia, por el Dr. Santiago Macías.
- Hospital Galdakao-Usansolo, por el Dr. Higinio Martín.
- Hospital virgen de la Arrixaca, por el Dr. Domingo Martínez.
- Hospital Universitario Puerta del Hierro Majadahonda, por el Dr. Miguel Ángel Romera.
- Hospital Universitario 12 de Octubre, por el Dr. Emilio alted.
- Hospital Universitario Severo Ochoa, por el Dr. Frutos del Nogal.

Lo que firmo en Valencia a 9 de noviembre de 2012.

Firmado:
Don Manuel Labiós Gómez

GENERALITAT VALENCIANA

AGÈNCIA VALENCIANA DE SALUT

D. Manuel Labiós Gómez, Secretario del Comité Ético de Investigación Clínica del Hospital Clínico Universitario de Valencia

CERTIFICA

Que, tal como consta en el Acta de la Comisión Permanente nº 87, de fecha 9 de noviembre de 2012 se hallan presentes los siguientes miembros del Comité:

Presidente:
Dr. D. A. Peláez Hernández — Jefe Servicio. Alergia

Vicepresidente:
Dr. D. J. Magraner Gil — Jefe Servicio. Farmacia

Secretario:
Dr. D. M. Labiós Gómez — Jefe Sección. Medicina Interna

Vocal:
Dr. D. J. Blanquer Olivas — Jefe Sección. Medicina Intensiva

Valencia, 9 de noviembre de 2012.

Fdo.: D. Manuel Labiós Gómez

DICTAMEN DEL COMITE ETICO DE INVESTIGACION CLINICA

Dr. D. Manuel Labiós Gómez Secretario del Comité Ético de Investigación Clínica del Hospital Clínico Universitario de Valencia

CERTIFICA
Que este Comité, en calidad de CEIC de referencia, en su reunión de 26 de marzo de 2015, acta 302, ha evaluado la propuesta del promotor relativa a la modificación:

Nº de modificación: Enmienda relevante nº 2 de fecha 11 de febrero de 2015

Versión previa de Protocolo: Versión 3.0 de 3 de Junio de 2012
Versión actual de Protocolo: Versión 3.0 de 3 de Junio de 2012de 2011

Perteneciente al Estudio:
Nº EUDRACT: 2012-000775-17
Código de protocolo del promotor: PI11-0143
Versión/fecha del protocolo: Versión 3.0 de 3 de Junio de 2012
Hoja de información al paciente/sujeto: Versión 2.0 de 16 de octubre de 2011
Título: "Estudio comparativo, aleatorio, controlado para evaluar la eficacia dde la administración de dexametasona en el tratamiento de pacientes con el Síndrome de Distrés Respiratorio Agudo"
Promotor:.Jesús Villar Hernández

Este Comité, emite un DICTAMEN FAVORABLE para la realización de la modificación al ensayo. Teniendo en cuenta los informes recibidos de los CEIC implicados, acepta su realización en los centros que se listan a continuación:

- Hospital Clínico Universitario de Valencia, por el Dr. Fco. Javier Belda.
- Hospital General Virgen de la Luz, por el Dr. José M. Añón.
- Hospital Universitario Rio Hortega, por el Dr. Jesús Blanco.
- Complejo Hospitalario Universitario de Santiago, por el Dr. Antonio Santos.
- Hospital José M. Morales Meseguer, por el Dr. Gumersindo González.
- Complejo Asistencial Universitario de León, por el Dr. Demetrio Carriedo.
- Complexo Hospitalario Universitario a Coruña, por el Dr. Fernando Mosteiro.
- Hospital General de Ciudad Real, por el Dr. Alfonso Ambrós.
- Hospital General La Mancha Centro, por la Dra. Carmen Martín.
- Hospital Universitari Mutua de Terrassa, por la Dra. Mª Mar Fernández.
- Hospital Moncloa, por el Dr. Vicente Gómez.
- Hospital Nuestra Señora de la Candelaria, por la Dra. Elena Espinosa.
- Hospital Universitario de Cruces, por el Dr. Fermín Labayen.
- Hospital General de Segovia, por el Dr. Santiago Macías.
- Hospital Galdakao-Usansolo, por el Dr. Higinio Martín.
- Hospital virgen de la Arrixaca, por el Dr. Domingo Martínez.
- Hospital Universitario Puerta del Hierro Majadahonda, por el Dr. Miguel Ángel Romera.
- Hospital Universitario 12 de Octubre, por el Dr. Emilio Alted.
- Hospital Universitario Severo Ochoa, por el Dr. Frutos del Nogal.
- Hospital Nuestra Señora del Prado, por el Dr. Francisco Alba.
- Hospital Virgen de la Concha, por la Dra. Ana Caballero.
- Hospital Virgen de la Salud, por la Dra. Mª del Mar Cruz

Lo que firmo en Valencia, a 10 de abril de 2015

Fdo. : Dr. D. Manuel Labiós Gómez
Secretario del Comité

Complejo Asistencial Universitario de León
Altos de Nava s/n. 24071 LEÓN. Tel. 987 237 400 Fax. 987 233 322

Sacyl

CONFORMIDAD DE LA DIRECCIÓN DEL CENTRO

D. Juan Luis Burón Llamazares, Director Gerente del Complejo Asistencial Universitario de León y vista la autorización del Comité Ético de Investigación Clínica.

CERTIFICA:

Que conoce la propuesta realizada por el Promotor D. JESÚS VILLAR HERNÁNDEZ DE LA UNIDAD DE INVESTIGACIÓN DEL HOSPITAL UNIVERSITARIO DE GRAN CANARIA "DR. NEGRÍN", para que sea realizado en este Centro el estudio titulado *"Estudio comparativo, aleatorio, controlado, para evaluar la eficacia de la administración de DEXAMETASONA en el tratamiento de pacientes con el Síndrome de Distrés Respiratorio Agudo"* **Código de Protocolo:** PI11-0143; **EudraCT** 2012-000775-17, desarrollado por el Dr. Demetrio Carriedo Ule del Servicio de Medicina Intensiva como investigador principal.

Que está de acuerdo con el contrato firmado entre el Centro y el promotor en el que se especifican todos los aspectos económicos del estudio.

Que acepta la realización de dicho estudio en este Centro.

Lo que firma en León a 12 de mayo de 2013

Fdo: Juan Luis Burón Llamazares
Director Gerente
Complejo Asistencial Universitario de León

201208+

CEIC E

EUSKO JAURLARITZA
GOBIERNO VASCO

OSASUN ETA KONTSUMO SAILA
DEPARTAMENTO DE SANIDAD Y CONSUMO

INFORME DEL COMITE ETICO DE INVESTIGACION CLINICA DE EUSKADI (CEIC-E)

Dra. Iciar Alfonso Farnós como Secretaria del CEIC de la Comunidad Autónoma de País Vasco (CEIC-E)

CERTIFICA

Que este Comité, que reúne los requisitos establecidos en el R.D 223/2004, de 6 de Febrero por el que se regulan los ensayos clínicos con medicamentos, ha evaluado la propuesta del promotor: Jesús Villar Hernández, para que se realice el ensayo clínico Código Promotor: **PI11-0143**, Nº EUDRACT: **2012-000775-17,** de Título: **"Ensayo clínico comparativo, aleatorio, controlado para evaluar la eficacia de la administración de dexametasona en el tratamiento de pacientes con Síndrome de Distrés Respiratorio Agudo"**

Versión del Protocolo: 3.0 de 03/06/2012
Versión Hoja Información al Paciente y Consentimiento Informado: GENERAL / 2.0 de 16/10/2012

Considera que,

Se cumplen los requisitos necesarios de idoneidad del protocolo en relación con los objetivos del estudio y están justificados los riesgos y molestias previsibles para el sujeto.

La capacidad de los Investigadores y los medios disponibles son apropiados para llevar a cabo el estudio.

Son adecuados tanto el procedimiento para obtener el consentimiento informado como la compensación prevista para los sujetos por daños que pudieran derivarse de su participación en el ensayo.

El alcance de las compensaciones económicas previstas no interfiere con el respeto a los postulados éticos.

Este Comité Ético de Investigación Clínica, tanto en su composición como en los Procedimientos normalizados de Trabajo, cumple con las normas de buena práctica clínica (CPMP/ ICH/135/95) conforme a lo establecido en el capítulo VIII del Real Decreto 223/2004, de 6 de Febrero.

Y que este Comité reunido el día 29/05/2013 (recogido en acta 05/2013) ha decidido emitir **informe favorable** a que dicho ensayo sea realizado por los siguientes Investigadores:

- Tomás Muñoz Martinez *(Cuidados Intensivos) Hospital Universitario Cruces;*
- Rosa Ana Rivas Gómez *(Cuidados Intensivos) Hospital de Galdakao*

Lo que firmo en Vitoria-Gasteiz, a 30 de Mayo de 2012

Fdo:

EUSKO JAURLARITZA
GOBIERNO VASCO
OSASUN SAILA
DEPARTAMENTO DE SALUD

30 MAY 2013

Euskadiko Ikerketa Klinikoetarako Batzorde Etikoa
Comité Ético de Investigación Clínica de Euskadi (CEIC-E)

Dra. Iciar Alfonso Farnós
Secretaria del CEIC de la Comunidad Autónoma de País Vasco (CEIC-E)

Hospital Universitario 12 de Octubre
SaludMadrid · Comunidad de Madrid

i+12 Instituto de Investigación Hospital 12 de Octubre

CONFORMIDAD DE LA DIRECCIÓN DEL CENTRO

D. FERNANDO MEJÍA ESTEBARANZ, Director Médico del Hospital Universitario 12 de Octubre de Madrid, y vista la autorización del Comité Ético de Investigación Clínica,

CERTIFICA

Que conoce la propuesta realizada por el Promotor ***JESUS VILLAR HERNANDEZ*** para que sea realizado en este Centro el ***ENSAYO CLINICO***, *número de CEIC:* ***12/201,***

Titulado: ***ESTUDIO COMPARATIVO, ALEATORIO, CONTROLADO, PARA EVALUAR LA EFICACIA DE LA ADMINISTRACIÓN DE DEXAMETASONA EN EL TRATAMIENTO DE PACIENTES CON EL SÍNDROME DE DISTRÉS RESPIRATORIO AGUDO,***

Con CODIGO DE PROTOCOLO: ***PI11-0143***

Y que será realizado por el Dr. ***Emilio ALTED LOPEZ,*** del Servicio/Unidad: ***URGENCIAS (INTENSIVOS)*** como Investigador Principal.

Que esta de acuerdo con el contrato firmado entre el Centro y el promotor en el que se especifican todos los aspectos económicos de este ***ENSAYO CLINICO***

Que acepta la realización de dicho ***ENSAYO CLINICO*** en este Centro.

Lo que firma en Madrid, a 26 de abril de 2013,

Fdo.: Fernando Mejía Estebaranz
DIRECTOR MEDICO

SaludMadrid Hospital Universitario 12 de Octubre Comunidad de Madrid
DIRECCIÓN MÉDICA

D. José Manuel Vázquez Rodríguez, Coordinador de Docencia, I+D+i del Área de Gestión Integrada de A Coruña, vista la autorización del Comité Ético de Investigación Clínica de Galicia y del Comité Ético de Investigación Clínica de Referencia.

CERTIFICA

Que conoce la propuesta realizada por el promotor **Dr. Jesús Villar Hernández** para que se lleve a cabo en el Servicio de Unidade Coidados Intensivos del Hospital Universitario A Coruña de la Xerencia de Xestión Integrada de A Coruña el ensayo clínico con código **DEXA** y número de EudraCT 2012-000775-17 titulado: '*Estudio comparativo, aleatorio, controlado para evaluar la eficacia de la administración de dexametasona en el tratamiento del Síndrome de Distrés Respiratorio Agudo*',que será realizado en este Centro por el Dr. Fernando Mosteiro Pereira como Investigador Principal.

Que está de acuerdo con el contrato firmado entre el Centro y el promotor en el que se especifican todos los aspectos económicos de este estudio clínico.

Que acepta la realización de dicho ensayo en este Centro

Lo que firma en A Coruña a 25 de noviembre de 2013

Xerencia de Xestión Integrada
Coordinador de Docencia, I+D+i

Fdo. D. José Manuel Vázquez Rodríguez
Coordinador Docencia e I+D+i
Gerencia de Gestión Integrada de A Coruña

Hospital General Universitario de Ciudad Real

sesca

C/ Obispo Rafael Torija, s/n
Telf. 926 27 80 00 - Fax. 926 27 85 02
13005 CIUDAD REAL

FECHA: 21 de Febrero de 2013

DE: DIRECCIÓN GERENCIA

A: CEIC

ASUNTO: Autorización SESCAM Ensayo Clínico

En relación con la solicitud para realizar el ensayo clínico, código PI11-0143, de título "**"*Estudio comparativo , aleatorio, controlado, para evaluar la eficacia de la administración de dexametasona en el tratamiento de pacientes con el Síndrome de Distrés Respiratorio Agudo*"** ", nº de eudract 2012-775-17 , cuyo promotor es el Dr. Jesús Villar Hernández , vista la documentación presentada y visto el informe favorable del Área de Farmacia del SESCAM, **te comunico la conformidad** de esta Dirección Gerencia para la realización del mismo.

No obstante en el contrato que se firme para su realización se deberán incluir las siguientes cláusulas:

- El promotor se compromete a publicar los resultados del estudio (art. 38-1 del RD 223/2004), al menos en una publicación científico-médica de ámbito nacional o internacional con revisores externos y referenciada en índices bibliográficos de publicaciones bio-médicas, y a citar en la misma al Servicio de Salud de Castilla-La Mancha entre los organismos participantes de este Servicio de Salud.

- En concordancia con el artículo 27 del Real Decreto 223/2004, una vez terminada la realización del ensayo, el promotor enviará al Área de Farmacia del SESCAM un resumen del informe final sobre los resultados del mismo. La información incluirá los datos correspondientes de seguridad de acuerdo con lo estipulado en el art. 47 del citado Real Decreto.

- El acceso a las historias clínicas que, de acuerdo con la normativa aplicable sobre Buena Práctica Clínica, pudiera efectuarse por parte del promotor, se efectuará en las condiciones que establezca cada centro sanitario donde se vaya a realizar el estudio garantizando, en todo caso, que los datos obtenidos no permitan identificar a la persona.

Hospital General Universitario de Ciudad Real

sescam

C/ Obispo Rafael Torija, s/n
Telf. 926 27 80 00 - Fax. 926 27 85 02
13005 CIUDAD REAL

- Los datos personales de pacientes que sean comunicados al promotor serán previamente disociados, de modo que la información que obtenga no pueda ligarse en modo alguno a persona identificada o identificable. El promotor o sus representantes deberán cumplir en todo momento la normativa vigente en materia de protección de datos de carácter personal.

- El número máximo de pacientes a incluir en el estudio será de 15. Cualquier variación en el número de participantes, conllevará en su caso, las consiguientes repercusiones económicas. Esta circunstancia se comunicará formal y fehacientemente, en caso de ser aceptada por la Dirección Gerencia del SESCAM, a los órganos competentes, así como al centro y a la entidad gestora, no siendo necesaria la formalización de cláusula o documento adicional al presente contrato.

En cuanto a la cuantía de las contraprestaciones económicas, deberá mantenerse el principio de que la contraprestación para el Centro, no sea inferior a la que corresponde al investigador.

Asímismo una vez fimado el correspondiente contrato, se comunicará a la citada Área de Farmacia del SESCAM.

Un saludo,

Fdo.: Ángel Gómez Roig
EL DIRECTOR GERENTE

Fundación Ramón Domínguez
I+D+i biosanitaria

CONFORMIDAD DE LA DIRECCIÓN DEL CENTRO

Dr. Luis Verde Remeseiro, Xerente de Xestión Integrada de Santiago de Compostela del Hospital Clínico de Santiago y vista la autorización del Comité Ético de Investigación Clínica de Galicia,

C E R T I F I C A

Que conoce la propuesta realizada por el promotor **Jesús Villar Hernandez del Hospital Universitario Dr. Negrín de Las Palmas de Gran Canarias** para que sea realizado en este Centro el ensayo clínico código de protocolo **PI 11-0143,** titulado "***Estudio comparativo, aleatorio, controlado, para evaluar la eficacia de la administración de dexametasona en el tratamiento de pacientes con el Síndrome de Distrés Respiratorio Agudo***" cuyo investigador principal es el **Dr. Antonio Santos Bouzas**, Jefe del Servicio de Medicina Intensiva del CHUS.

Que está de acuerdo con la propuesta de contrato presentada al Centro por el promotor, en el que se especifican todos los aspectos económicos de este ensayo clínico.

Que acepta la realización de dicho ensayo clínico en este Centro.

Lo que firma en Santiago, a 14 de junio de 2013.

Fdo.:
Dr. Luis Verde Remeseiro

Informe Dictamen Protocolo Favorable
EECC con Medicamento – Multicéntrico

C.P. PI11-0143 - N.E. 2012-000775-17 - C.I. EN-18/12

24 de octubre de 2012

Dra. María Dolores Nájera Pérez
Presidenta del CEIC Hospital General Universitario José María Morales Meseguer

CERTIFICA

1º. Que el CEIC Hospital General Universitario José María Morales Meseguer en su reunión del día 24/10/2012, acta ORDINARIA ha evaluado la propuesta del promotor referida al estudio:

Título: Estudio comparativo, aleatorio, controlado, para evaluar la eficacia de la administración de dexametasona en el tratamiento de pacientes con el Síndrome de Distrés Respiratorio Agudo.

Código Promotor: PI11-0143 **Nº EUDRACT:** 2012-000775-17 **Código Interno:** EN-18/12
Promotor: Otros (académico)
Monitor/CRO: Otros (académico)
Versión Protocolo Evaluada: 3.0 de 3 Junio 21012
Versión Hoja Información al Paciente Evaluada: 2.0 16/10/2012
Fecha Entrada: 05/07/2012

2º. Considera que:
- El ensayo se plantea siguiendo los requisitos del Real Decreto 223/2004, de 6 de febrero y las normas que lo desarrollan, y su realización es pertinente.
- Se cumplen los requisitos necesarios de idoneidad del protocolo en relación con los objetivos del estudio y están justificados los riesgos y molestias previsibles para el sujeto.
- Son adecuados tanto el procedimiento para obtener el consentimiento informado como la compensación prevista para los sujetos por daños que pudieran derivarse de su participación en el ensayo.
- El alcance de las compensaciones económicas previstas no interfiere con el respeto a los postulados éticos.
- La capacidad del investigador y sus colaboradores, y las instalaciones y medios disponibles, tal y como ha sido informado, son apropiados para llevar a cabo el estudio.

3º Por lo que este CEIC emite un **DICTAMEN FAVORABLE.**
4º Este CEIC acepta que dicho ensayo sea realizado en los siguientes CEIC/Centros por los Investigadores:

CEIC Hospital General Universitario José María Morales Meseguer	Dr. Andres Carrillo Alcaraz *(Medicina Intensiva) Hospital General Universitario J.M. Morales Meseguer*<br>Dr. Juan Alfonso Soler Barnes *(Medicina Intensiva) Hospital General Universitario J.M. Morales Meseguer*

Consejería de Sanidad y Consumo · Región de Murcia · Servicio Murciano de Salud · Hospital J. Mº Morales Meseguer · Murcia · Comité Ético de Investigación Clínica

Lo que firmo en Murcia, a 24 de octubre de 2012

Dra. María Dolores Nájera Pérez.
Presidenta del CEIC Hospital General Universitario José María Morales Meseguer

Hospital General Universitario J.M. Morales Meseguer
Marqués de los Vélez s/n Murcia 30008 Murcia España
Tel. 968 36 09 89 Fax. 968 36 09 49 Correo electrónico mariad.najera@carm.es

"Hospital Morales Meseguer" – C/. Marqués de los Vélez, s/n • Teléf. 968 36 09 00 • Fax. 968 23 24 84 • 30008 MURCIA

Virginia Arroyo Pineda, Secretaria del Comité Ético de Investigación Clínica del Área Integrada de Salud de Talavera de la Reina,

CERTIFICA

Que este Comité en las reuniones celebradas entre el día 19 de febrero y 16 de abril de 2.013 ha evaluado la propuesta referida al estudio:

- **Título:** Estudio comparativo, aleatorio para evaluar la eficacia de la administración de Dexametasona en el tratamiento del síndrome de distrés respiratorio agudo.
- **Protocolo nº:** PI11-0143.
- **Promotor:** Jesús Villar Hernández.
- **Tipo de estudio:** Ensayo Clínico.
- **Investigador en el área**: Dr. Francisco Alba (Medicina Intensiva de la UCI).

Considera que:

- Se cumplen los requisitos necesarios de idoneidad del proyecto en relación con los objetivos del mismo.
- Que la capacidad del investigador y los medios disponibles son apropiados para llevar acabo el estudio.
- Que no interfiere los postulados éticos y respeta los derechos fundamentales de la persona.

Por lo que emite un **DICTAMEN FAVORABLE**.

Lo que firmo en Talavera de la Reina, a 16 de abril de 2.013.

Servicio de Salud de Castilla la Mancha · sescam · CEIC · Área Integrada de Talavera de la Reina

Fdo: Virginia Arroyo Pineda.
Secretaria del CEIC

CEIC del AGI de Talavera de la Reina
Hospital Nuestra Señora del Prado - Ctra. Nacional V, km. 114
Talavera de la Reina 45600 Toledo España
Tel. (925) 80 36 00 Ext. 86.316; varroyo@sescam.org

Hospital Universitario Puerta de Hierro Majadahonda
SaludMadrid
Comunidad de Madrid

Comunidad de Madrid

CONFORMIDAD DE LA DIRECCIÓN DEL CENTRO

*Doña **María Codesido López**, Directora Gerente del **Hospital Universitario Puerta de Hierro Majadahonda**, y vista la autorización del Comité Ético de Investigación Clínica,*

CERTIFICA

*Que conoce la propuesta realizada por el promotor **Dr. Jesús Villar Hernández,** para que sea realizado en este centro el ensayo clínico con código de protocolo **PI 11-0143**, titulado: **"Estudio comparativo, aleatorio, controlado para evaluar la eficacia de la administración de dexametasona en el tratamiento del Síndrome de Distrés Respiratorio Agudo"**. Nº Eudract: **2012-000775-17**. Protocolo Versión **3.0 de 3 de junio de 2012.** HIP/CI Versión **2.0 de 16/10/11**, que será realizado por el **Dr. Miguel Ángel Romera**, perteneciente al **Servicio de Medicina Intensiva** como Investigador Principal.*

Que considera idóneos al investigador principal y colaboradores, así como las instalaciones en las que el ensayo va a realizarse.

En cualquier caso, la realización del presente ensayo clínico quedará supeditada a la firma previa de un contrato entre el promotor, investigador principal y el Presidente de la Fundación para la Investigación Biomédica del centro.

Que acepta la realización de dicho ensayo clínico en este centro.

Lo que firmo en Majadahonda, a 16 de Abril de 2013

Hospital Universitario Puerta de Hierro Majadahonda

*Fdo: **Dña. María Codesido López***

CONFORMIDAD DE LA DIRECCIÓN DEL CENTRO

D. JOSE MIGUEL GARCIA VELA, Director Médico del Hospital Universitario del Río Hortega de Valladolid y vista la autorización del Comité Ético de Investigación Clínica.

CERTIFICA:

Que esta Dirección ha conocido la propuesta del **Promotor D. Jesús Villar Hernández** para que se realice el Ensayo Clínico titulado:**"Estudio comparativo, aleatorio, controlado, para evaluar la eficacia de la administración de dexametasona en el tratamiento de pacientes con el Síndrome de Distrés Respiratorio Agudo"**
Código de Protocolo PI11-0143. Eudract 2012-000775-17
Investigador Principal: Dr. J. Blanco Varela

Que está de acuerdo con el contrato firmado entre el Centro y el Promotor en el que se especifican todos los aspectos económicos del mismo.

Que acepta la realización de dicho Proyecto de Investigación en este Centro.

Lo que firma en Valladolid, a 26 de Julio de 2012

JUNTA DE CASTILLA Y LEÓN
GERENCIA REGIONAL
DIRECCIÓN MÉDICA
SACYL
HOSPITAL UNIVERSITARIO RÍO HORTEGA

Fdo. Dr. D. José Miguel García Vela
Director Médico

Fundación Ramón Domínguez
I+D+i biosanitaria

CONFORMIDAD DE LA DIRECCIÓN DEL CENTRO

Dr. Luis Verde Remeseiro, Xerente de Xestión Integrada de Santiago de Compostela del Hospital Clínico de Santiago y vista la autorización del Comité Ético de Investigación Clínica de Galicia,

C E R T I F I C A

Que conoce la propuesta realizada por el promotor **Jesús Villar Hernandez Hospital Universitario Dr. Negrín de Las Palmas de Gran Canarias** para que sea realizado en este Centro el ensayo clínico código de protocolo **PI 11-0143,** titulado "***Estudio comparativo, aleatorio, controlado, para evaluar la eficacia de la administración de dexametasona en el tratamiento de pacientes con el Síndrome de Distrés Respiratorio Agudo***" cuyo investigador principal es el **Dr. Julián Alvarez Escudero**, Jefe del Servicio de Anestesia y Reanimación del CHUS.

Que está de acuerdo con la propuesta de contrato presentada al Centro por el promotor, en el que se especifican todos los aspectos económicos de este ensayo clínico.

Que acepta la realización de dicho ensayo clínico en este Centro.

Lo que firma en Santiago, a 13 de junio de 2013.

Fdo.:

Dr. Luis Verde Remeseiro

Servicio
Murciano
de Salud

Arrixaca
Hospital Universitario
"Virgen de la Arrixaca"
Ctra. Madrid - Cartagena • Telf. 968 36 95 00
30120 El Palmar (Murcia)

JOSE VICENTE ALBALADEJO ANDREU, DIRECTOR GERENTE DEL ÁREA I DE SALUD MURCIA-OESTE, HOSPITAL CLÍNICO UNIVERSITARIO VIRGEN DE LA ARRIXACA,

EXPONE

1.- Que conoce la propuesta hecha por D. Jesús Villar Hernández, -Unidad de Investigación del Hospital Universitario de Gran Canaria Dr. Negrín, en calidad de Promotor, para que sea realizado en este Hospital el "Estudio comparativo, aleatorio, controlado, para evaluar la eficacia de la administración de dexametasona en el tratamiento de pacientes con el Síndrome de Distrés Respiratorio Agudo", por el **Dr. D. Domingo Martínez Baño,** -Servicio de Medicina Intensiva- como Investigador Principal.

2.- El estudio ha sido evaluado favorablemente por el Comité Ético de Investigación Clínica del Hospital en su reunión de 24 de septiembre de 2012.

2.- Que en consecuencia con lo anterior, ésta Dirección Gerencia acepta y presta su conformidad para que el estudio antes referido se realice en el Hospital Clínico Universitario Virgen de la Arrixaca.

Murcia, 8 de marzo de 2013.

COMUNIDAD AUTONOMA DE LA REGION DE MURCIA
CONSEJERIA DE SANIDAD Y CONSUMO
Servicio Murciano de Salud
Hospital "Virgen de la Arrixaca"
DIRECCIÓN GERENCIA

Mod. 141

sescam
Servicio de Salud de Castilla-La Mancha

CASTILLA-LA MANCHA
REGISTRO INTERNO
CONSEJERÍA DE SANIDAD Y ASUNTOS SOCIALES
Servicio de Salud de Castilla-La Mancha - TOLEDO
14 FEB 2013
Anotación N.º 030792

Dirección-Gerencia

Toledo, 12 de febrero de 2013

Sr. D.
Rodolfo Antuña Pellegri
Director Gerente del Hospital "Virgen de la Luz"
Hermandad de Donantes de Sangre, 1
CUENCA

Estimado Rodolfo:

En relación con la solicitud para realizar un estudio clínico, de título *"Estudio comparativo, aleatorizado, controlado para evaluar la eficacia de la administración de dexametasona en el tratamiento de pacientes con el Síndrome de Distrés Respiratorio Agudo"*, nº de eudract 2012-000775-17, código PI11-0143, cuyo promotor es el Dr. Jesús Villar Hernández, vista la documentación presentada y visto el informe favorable del Área de Farmacia del SESCAM, **te comunico la conformidad** de esta Dirección Gerencia para la realización del mismo.

No obstante, en el contrato que se firme para su realización se deberán incluir las siguientes cláusulas:

- El promotor se compromete a publicar los resultados del estudio (art. 38-1 del RD 223/2004), al menos en una publicación científico-médica de ámbito nacional o internacional con revisores externos y referenciada en índices bibliográficos de publicaciones bio-médicas, y a citar en la misma al Servicio de Salud de Castilla-La Mancha entre los organismos participantes en dicho estudio, así como a los investigadores participantes de este Servicio de Salud.
- En concordancia con el artículo 27 del Real Decreto 223/2004, una vez terminada la realización del ensayo, el promotor enviará al Área de Farmacia del SESCAM un resumen del informe final sobre los resultados del mismo. La información incluirá los datos correspondientes de seguridad de acuerdo con lo estipulado en el art. 47 del citado Real Decreto.
- El acceso a las historias clínicas que, de acuerdo con la normativa aplicable sobre Buena Práctica Clínica, pudiera efectuarse por parte del promotor, se efectuará en las condiciones que establezca cada centro sanitario donde se vaya a realizar el estudio garantizando, en todo caso, que los datos obtenidos no permitan identificar a la persona.

sescam
Servicio de Salud de Castilla-La Mancha

Dirección-Gerencia

- Los datos personales de pacientes que sean comunicados al promotor serán previamente disociados, de modo que la información que obtenga no pueda ligarse en modo alguno a persona identificada o identificable. El promotor o sus representantes deberán cumplir en todo momento la normativa vigente en materia de protección de datos de carácter personal.

- El número máximo de pacientes a incluir en el ensayo será de 15. Cualquier variación en el número de participantes, conllevará en su caso, las consiguientes repercusiones económicas. Esta circunstancia se comunicará formal y fehacientemente, en caso de ser aceptada por la Dirección Gerencia del SESCAM, a los órganos competentes, así como al centro y a la entidad gestora, no siendo necesaria la formalización de cláusula o documento adicional al presente contrato.

En cuanto a la cuantía de las contraprestaciones económicas, deberá mantenerse el principio de que la contraprestación para el Centro no sea inferior a la que corresponde al investigador.

Asimismo, una vez firmado el correspondiente contrato, se comunicará a la citada Área de Farmacia del SESCAM.

Un saludo,

EL DIRECTOR GERENTE

CONSEJERÍA DE SANIDAD Y ASUNTOS SOCIALES
Castilla-La Mancha
sescam

Fdo.: Luis Carretero Alcántara

COMPLEJO HOSPITALARIO DE TOLEDO
HOSPITAL VIRGEN DE LA SALUD
Avda. Barber, 30. 45004. Toledo. Teléfono 925 269200

sescam

CONFORMIDAD DE LA DIRECCIÓN DEL CENTRO

Dª CRISTINA GRANADOS ULECIA, Directora Gerente del Complejo Hospitalario de Toledo y vista la autorización del Comité Ético de Investigación Clínica,

CERTIFICA

Que conoce la propuesta realizada por el promotor: D. Jesús Villar Hernández, para que sea realizado en este Complejo Hospitalario de Toledo el **ENSAYO CLÍNICO** de título: "Estudio comparativo, aleatorio, controlado para evaluar la eficacia de la administración de dexametasona en el tratamiento de pacientes con el Síndrome de Distrés Respiratorio Agudo", que será desarrollado por el/la investigador/a:

Dª Mª DEL MAR CRUZ ACQUARONI.

Que está de acuerdo con el contrato firmado entre el Centro y el Promotor de fecha 26/MAYO/2015, en el que especifican todos los aspectos económicos del **ENSAYO, que en el presente contrato no tiene contraprestación económica**.

Que acepta la realización de dicho **ENSAYO** en este Centro.

Lo que firma en Toledo, a 13 de julio de 2015.

LA DIRECTORA GERENTE DEL C.H.T

Servicio de Salud de Castilla-La Mancha
COMPLEJO HOSPITALARIO
sescam
DIRECCIÓN GERENCIA
TOLEDO

Fdo.: Cristina Granados Ulecia
