# Supplementary material for: Evaluating the efficacy of dexamethasone in the treatment of patients with persistent acute respiratory distress syndrome: study protocol for a randomized controlled trial
Source: Trials. 2016 Jul 22;17:342. doi: 10.1186/s13063-016-1456-4 (PMC4957909; doi:10.1186/s13063-016-1456-4)
Supplement: Additional file 2: — Informed consent form. (PDF 114 kb) [file 13063_2016_1456_MOESM2_ESM.pdf]

Estudio comparativo, aleatorio, controlado para evaluar la eficacia de la administración de dexametasona en el tratamiento de pacientes con el Síndrome de Distrés Respiratorio Agudo

Consentimiento informado

Propuesta de participación en un estudio de investigación médica

Usted (o su familiar) tiene un Síndrome de Distrés Respiratorio Agudo (SDRA), la principal causa de morbilidad y mortalidad en pacientes con sepsis, neumonía, traumatismos graves y otras enfermedades pulmonares y sistémicas que sufren muchos pacientes ingresados en las Unidades de Medicina Intensiva. Causado por un proceso inflamatorio intenso e incontrolado, se produce como consecuencia de una lesión pulmonar aguda grave que se caracteriza por la formación de edema pulmonar, incapacidad para mantener la función respiratoria normal e inadecuada oxigenación de la sangre.

Aunque en los últimos 20 años hemos aumentado mucho nuestro conocimiento sobre los mecanismos que causan el SDRA y los factores que determinan su evolución, hasta la fecha no existe un tratamiento farmacológico específico. El único tratamiento que existe es la ventilación mecánica con aire enriquecido con oxígeno, el mismo tratamiento que está recibiendo en estos momentos. Sin embargo, a veces, las presiones que origina el respirador mecánico y las altas concentraciones de oxígeno que está recibiendo no están exentas de daño, sobre todo en personas con una especial predisposición genética a tener una respuesta inflamatoria exagerada.

Dado que el SDRA se caracteriza fundamentalmente por una intensa reacción inflamatoria en los pulmones, los corticoides deberían ser el tratamiento ideal para disminuir el daño pulmonar dado el potente efecto anti-inflamatorio que todos ellos tienen. En la mayoría de las Unidades de Medicina Intensiva, los pacientes con infecciones y traumatismos graves suelen recibir corticoides para intentar atenuar la exagerada respuesta anti-inflamatoria e impedir que se propague por todo el organismo.

Algunos estudios médicos realizados recientemente por investigadores norteamericanos y europeos en pacientes que tienen SDRA, como Vd. o su familiar, han observado que los corticoides pueden ser beneficiosos para mejorar la función pulmonar y la supervivencia en pacientes con neumonía bacteriana grave y en pacientes con SDRA cuando se administra antes de los 14 días.

Queremos proponerle participar en un estudio científico para comprobar y demostrar que si añadimos de forma temprana un tratamiento con corticoides a su tratamiento general, podemos limitar la respuesta inflamatoria exagerada de sus pulmones y facilitar su recuperación. Esta investigación se realizará en varios hospitales de España con la participación de pacientes como usted (o su familiar) ingresados en la Unidad de Medicina Intensiva de este hospital y de otros hospitales españoles.

¿Por qué esta Usted siendo invitado/a a participar en este estudio de investigación médica?

Con este estudio pretendemos acelerar la recuperación de la lesión pulmonar aguda que se ha producido en sus pulmones como consecuencia de la enfermedad que ha motivado que sea tratado en estos momentos con un respirador mecánico y altas concentraciones de oxígeno.

Esta investigación se realizará en varios hospitales de España y estará coordinada por médicos expertos nacionales e internacionales en el campo de la insuficiencia respiratoria, el síndrome de distrés respiratorio agudo y la ventilación mecánica. Usted ha sido invitado a participar porque a través de los síntomas y signos de su enfermedad, cumple las características en las que la administración de corticoides a su tratamiento podría disminuir la inflamación de sus pulmones y acelerar su recuperación y retirada de la ventilación mecánica.

¿Qué me harán si participo?

El principal procedimiento involucrado es la ventilación mecánica, la cual ya la tiene usted (o su familiar) porque la necesita por su enfermedad y no por este estudio.

Lo único que añadiremos (con su consentimiento) a la medicación que reciba por orden facultativa será una dosis diaria durante 10 días (o hasta que no necesite el respirador, si es antes de 10 días) de dexametasona, un corticoide con efectos anti-inflamatorios potentes y muy conocidos, que se viene administrando de forma rutinaria desde hace más de 30 años en pacientes que tienen la misma enfermedad que usted (o su familiar) y en otras enfermedades más graves para el tratamiento de la inflamación aguda e intensa en varios órganos (cerebro, pulmón, hígado, riñón).

¿Hay beneficios al participar en esta investigación?

Basado en recientes conocimientos y observaciones, parece que la administración de corticoides de forma temprana podría ser de gran beneficio contra la inflamación pulmonar de pacientes que tienen una lesión pulmonar aguda como la suya (o de su familiar).

No podemos garantizar completamente que obtenga beneficios clínicos directos por su participación en este estudio, aunque el tratamiento con dexametasona a las dosis que será tratado no conlleva ningún riesgo añadido a la ventilación mecánica ni al tratamiento que está recibiendo.

¿Cuáles son los riesgos generales de participar en este estudio?

Los efectos secundarios de la dexametasona son muy raros a las dosis contempladas en este estudio. Las potenciales desventajas por el uso de corticoides deben ser valoradas frente a los potenciales beneficios. Con las dosis y la duración del tratamiento que se especifica en este estudio, serán extremadamente raros los episodios de hiperglucemia transitoria (como ocurre con otros corticoides).

En todo caso el promotor del estudio dispone de una póliza de seguros, de la compañía Zurich, que se ajusta a la legislación vigente y que le proporcionará la compensación e indemnización en caso de daño a su salud o de lesiones que pudieran producirse en relación con su participación en el estudio.

Queremos además informarle que ni los médicos investigadores, ni sus familiares, ni usted, ni nadie involucrado en sus cuidados médicos recibirán ningún tipo de honorarios ni recompensa económica por participar en este estudio.

Asignación al tratamiento

Dado que queremos saber si la duración de la ventilación mecánica bajo tratamiento con dexametasona es igual o menor que en el caso de que no se administrara este medicamento, no le garantizamos que aunque acepte participar en el estudio se le administre la medicación.

Queremos que entienda que dada la naturaleza aleatoria del estudio, usted (o su familiar) tienen un 50% de probabilidades de recibir la dexametasona como tratamiento suplementario al que ya recibe. La decisión de recibir la dexametasona no depende de los médicos que le tratan sino de un proceso de selección al azar, siguiendo instrucciones contenidas en un sobre cerrado del que ninguno de los investigadores tiene conocimiento previo del contenido.

Compromiso de información de datos relevantes

Los investigadores de este estudio se comprometen a informarle a usted o a sus familiares, a los médicos a su cargo de cualquier dato relevante que condicione su permanencia en el estudio.

¿Cuánto durará la investigación?

La duración del tratamiento es de 10 días, o menos (si sus pulmones no necesitan la ayuda del respirador).

¿Habrá privacidad y confidencialidad?

Su identidad quedará protegida de acuerdo con la Ley Orgánica de Protección de Datos de Carácter Personal (LOPD 15/1999). De acuerdo también con esta Ley LOPD 15/1999 usted (o su familiar o representante legal) tiene derecho de acceso, cancelación, rectificación y oposición a los Datos en cualquier momento. Las únicas personas que tendrán conocimiento de que está participando en este estudio serán usted (y/o sus familiares o su representante legal), los miembros del equipo de investigación, los miembros del Comité de Ética del Hospital y si es conveniente sus médicos y enfermeras mientras esté ingresado/a en la Unidad de Cuidados Críticos. Ninguna información sobre usted será revelada a otros sin su autorización por escrito. Cuando los resultados del estudio sean publicados o discutidos en Conferencias y Congresos, no se incluirá ninguna información que pudiera revelar tu identidad.

¿Puedo retirarme del estudio una vez empezado?

Su participación en este estudio es VOLUNTARIA. Si decide no participar, eso no afectará de ningún modo la atención que reciba en el hospital. Si decide participar, es libre de cambiar de idea en cualquier momento sin que ello afecte la atención médica y personal que reciba en la Unidad. La retirada del estudio significaría la interrupción de la administración de dexametasona y el cese del registro de los datos para investigación.

¿A quién debo contactar en caso de dudas?

Los médicos investigadores que conducen este estudio en este Hospital son ………………………………………………………………Puede expresar sus dudas ahora o cuando lo desee. Los médicos y las enfermeras le pondrán en contacto con ellos si no se encontraran en la Unidad.

¿Cumple este estudio con la normativa vigente?

Este estudio ha sido aprobado por el Comité Ético de Investigación del Hospital y respeta los principios fundamentales establecidos en la Declaración de Helsinki, el Convenio del Consejo de Europa relativo a los derechos humanos y la biomedicina, y en la Declaración Universal de la UNESCO sobre derechos humanos.

DECLARACIÓN Y FIRMA de consentimiento para la inclusión en el estudio comparativo, aleatorio, controlado para evaluar la eficacia de la administración de dexametasona en el tratamiento de pacientes con el Síndrome de Distrés Respiratorio Agudo

El Dr/Dra………………………………………………………………………………., médico especialista del Hospital……………………………………………………………………… me ha informado personalmente y de forma comprensible, dispuesta y satisfactoria para mí, sobre los términos en los que se va a desarrollar el estudio clínico, sus objetivos, y la ausencia de riesgos o complicaciones añadidas a los propios de la ventilación mecánica.

He tenido la oportunidad de leer la información suministrada en este consentimiento, así como exponer mis dudas y ha respondido de forma satisfactoria a todas mis preguntas y observaciones, comprendiendo la naturaleza y objetivos que se pretenden con este estudio (qué es, cómo re realiza y para qué sirve).

Como representante legal del paciente D/Dña……………………………………………………………….

Con DNI ………………..…., en calidad de………………………………….(parentesco o representación legal), he recibido la información completa en todos los términos anteriormente expresados, y doy el **CONSENTIMIENTO para la inclusión en el Estudio comparativo, aleatorio, controlado para evaluar la eficacia de la administración de dexametasona en el tratamiento de pacientes con el Síndrome de Distrés Respiratorio Agudo**
al paciente D/Dña……………………………..……………………………………………

Para que conste, firmo el presente documento después de haberlo leído.

……………………………..a………de……………………………de…………..

Firmado: ………………………………………………………..
Firma del Paciente o Representante Legal

____
Firma del Investigador

____
Nombre y Firma del Testigo (si es requerido)
